# Environment specific substitution tables for thermophilic proteins

**DOI:** 10.1186/1471-2105-8-S1-S15

**Published:** 2007-03-08

**Authors:** K Mizuguchi, M Sele, MV Cubellis

**Affiliations:** 1 Department of Biochemistry, University of Cambridge, UK; 2 Department of Applied Mathematics and Theoretical Physics, University of Cambridge, UK; 3 Dipartimento di biologia strutturale e funzionale, Universita' di Napoli"Federico II", Italy; 4 Current address: National Institute of Biomedical Innovation, Japan

## Abstract

**Background:**

Thermophilic organisms are able to live at high temperatures ranging from 50 to > 100°C. Their proteins must be sufficiently stable to function under these extreme conditions; however, the basis for thermostability remains elusive. Subtle differences between thermophilic and mesophilic molecules can be found when sequences or structures from homologous proteins are compared, but often these differences are family-specific and few general rules have been derived. The availability of complete genome sequences has now made it feasible to perform a large-scale comparison between mesophilic and thermophilic proteins, the latter of which primarily come from archaeal genomes although a few complete genomes of thermophilic eubacteria are also available.

**Results:**

We compared mesophilic proteins with their thermophilic counterparts of archaeal or eubacterial origins independently. This was based on the assumption that in these two kingdoms, different mechanisms may have been exploited for the adaptation of proteins at high temperatures. We derived the environment specific amino acid compositions of thermophilic proteins from 10 archaeal and seven eubacterial genomes, by aligning a large number of sequences from thermophilic proteins with their close mesophilic homologues of known three-dimensional (3D) structure. We further analysed environment specific substitutions, which lead from mesophilic proteins to either archaeal or eubacterial thermophilic proteins.

**Conclusion:**

Our comparisons were based on homology-based structural predictions for a large number of thermophilic proteins. We demonstrated that thermal adaptation in the archaeal and eubacterial kingdoms is achieved in different ways. The main differences concern the usage of Gln, Ile and positively charged amino acids. In particular archaeal organisms appeared to have acquired thermostability by substituting non-charged polar amino acids (such as Gln) with Glu and Lys, and non-polar amino acids with Ile on the surface of proteins.

## Background

Thermophilic organisms are able to live at high temperatures ranging from 50 to > 100°C. They belong either to the archaeal or the eubacterial kingdom and they have been subdivided, setting a somewhat arbitrary temperature boundary, into thermophiles and hyperthermophiles. Initially, most archaeal species were isolated from extreme habitats but it has recently become clear that archaea, as well as eubacteria, are widespread and abundant in several diverse niches [1].

Thermophilic organisms are interesting for several reasons and in particular because they are a source of very stable proteins. Understanding the higher-temperature resistance of thermophilic proteins is essential for the studies of protein folding and stability, and is critical for designing efficient enzymes that can work at high temperatures. Although many studies have been carried out for several decades, it has so far been difficult to identify any single factor as being primarily responsible for enhancing thermal stability. This is probably because protein stability is determined by a fine balance between several contributing factors. Moreover, even considering multiple factors, few general rules have been derived and often rules derived for one protein family did not apply to other families.

At least four different approaches have been used to study the stability of thermophilic proteins: 1) comparing a single thermophilic protein structure with its mesophilic homologues [2-7]; 2) modifying protein stability by mutagenesis [8-10]; 3) comparing datasets of high quality structures from thermophiles and mesophiles [11-13]; and 4) analysing whole genome sequences [14-17].

The analysis of high quality structures would be the most informative approach if a large dataset were available, but unfortunately this is not the case. On the other hand, the analysis of whole genomes can benefit from the increasing availability of large numbers of protein sequences. It is, therefore, desirable to combine the advantages of both approaches. This can be achieved by aligning sequences from thermophilic proteins with their close mesophilic homologues of known 3D structure. Since structure is better conserved than sequence, the alignment of thermophilic sequences to a homologous structure implies a likely 3D mapping of the protein sequences in question, yielding homology-based structural predictions for many proteins [18].

Aided by the recent progress in genome sequencing, we compared, in this paper, mesophilic proteins with archaeal and eubacterial thermophilic proteins separately. In doing so, we were motivated by the consideration that different strategies for thermal adaptation might have been exploited by organisms evolutionarily distant and that merging results obtained from thermophilic archaea and eubacteria might have hindered the previous attempts to identify the determinants of protein stability. We derived new general rules for thermal adaptation, specific to archaea and eubacteria.

## Results and discussion

### Databases

Two protein databases were created for organisms living above 50°C. One included 19,168 protein sequences derived from the genomes of 10 archaea, the other 17,040 protein sequences from the genomes of seven eubacteria. In Table 1 we report the names of the organisms, the temperature at which they live (OGT) and the GC content of their genomes.  Hyperthermophiles, i.e., organisms that live above 80°C are more frequently found in the archaeal kingdom. However, GC content does not correlate with OGTs and on average, is only slightly higher in eubacteria than in archaea.

A set of 3763 protein structures belonging to 1057 different families were taken from HOMSTRAD, a database of protein structural alignments for homologous families [19]. The sequence corresponding to each structure was used as a query to search separately against the two databases of thermophilic proteins. We used BLAST [20] under stringent conditions and detected close archaeal homologues of 1005 HOMSTRAD proteins and close eubacterial homologues of 1580 HOMSTRAD proteins. Accordingly we built 1005 alignments for archaea and 1580 alignments for eubacteria, where the first sequence from a mesophilic protein is aligned against its thermophilic homologues. The residues of the first protein, the structure of which is known, were assigned to one of eight different structural environments; alpha helix, exposed (HA) or buried (Ha), beta strand, exposed (EA) or buried (Ea), positive main-chain phi angle, exposed (PA) or buried (Pa) and coil, exposed (CA) or buried (Ca). We counted how many times an amino acid from the mesophilic sequence in a given environment is substituted by another amino acid in the thermophilic sequences (or is conserved). The total number of substitution counts was 3,011,344 for archaea and 4,432,631 for eubacteria.

For a comparison, we used alignments of mesophilic proteins stored in HOMSTRAD and counted how many times an amino acid from the mesophilic sequence in a given environment is substituted by another amino acid in homologous mesophilic sequences (or is conserved).

### Amino acid composition

**Figure 1:**
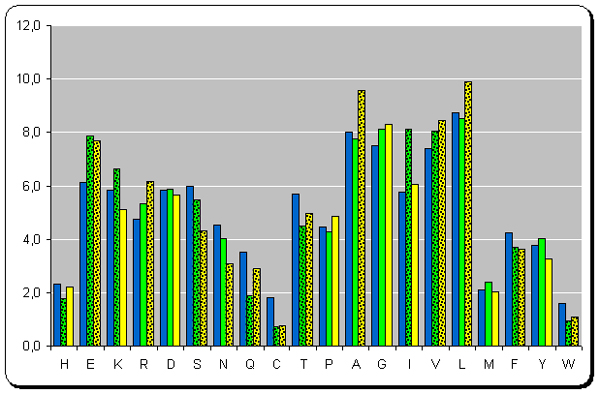
**Amino acid composition in percent**. Bars in blue are for mesophilic, in green for thermophilic archaeal, and in yellow for thermophilic eubacterial proteins. Dots indicate values that significantly differ (P < 0.01) between thermophilic and mesophilic proteins.

We counted the occurrence of each amino acid to derive the compositions of thermophilic proteins (of archaeal and eubacterial origins) and compared them with that of mesophilic proteins (Fig. 1) using a modified version of SUBST (K. Mizuguchi, unpublished).

Charged amino acids, more precisely, Lys in eubacteria, Arg in archaea, and Glu in both kingdoms, are more abundant in thermophilic proteins than in their mesophilic counterparts. Interestingly, Asp and His are not more abundant in either thermophilic group. As already reported [14,16,17], in thermophilic proteins the higher percentage of charged amino acids is compensated by the lower percentage of polar, non-charged amino acids (Ser, Thr, Asn, and Gln). However, we observed subtle differences between eubacteria and archaea; compared to mesophiles, Asn and Ser are significantly under-represented only in thermophilic eubacteria. Gln is under-represented in both eubacteria and archaea but more strongly in archaea.

It was proposed that Asn and Gln are avoided in thermophilic proteins because of their chemical instability at high temperatures due to deamidation [16]. Yet the deamidation of proteins occurs primarily at Asn residues, except in very long-lived proteins, where Gln deamidation is also observed [21]. If the chemical instability at high temperatures were the sole cause of avoiding Asn and Gln, Asn should be under-represented more strongly than Gln in both eubacterial and archaeal thermophilic proteins. The observed under-reprentation of Asn (and Ser) only in eubacteria and that of Gln only in archaea requires an alternative explanation.

These differences may be explained by the proposal that processes other than selection due to biochemical properties of the amino acids affect the patterns of amino substitution between mesophiles and thermophiles [22].  In addition to biochemical properties and the G/C content of their codons, amino acids differ in their cost of uptake, synthesis or incorporation into proteins. If these bioenergetic costs vary among domains, different patterns of amino acid substitutison can be observed between different pairs of mesophiles and thermophiles.

The under-representation of Gln in archaea is consistent with its bioenergetics. Glutaminyl-tRNA synthase is absent in archaea but is present in some eubacteria, while asparaginyl-tRNA synthase is absent in some eubacteria and archaea. In the organisms without Gln- and Asn-tRNA synthases, the inclusion of Asn and Gln into proteins involves the formation of mis-acylated Asp-tRNA(Asn) or Glu-tRNA(Gln), and their subsequent amidation catalysed by amidotransferases [23]. In thermophilic archaea, which lack Gln-tRNA synthases, Gln appears to be under-represented because of its instability at high temperatures and the cost of incorporating it into proteins. However, the previously reported negative correlation between the content of Gln and OGTs [24] still poses a question. The list of complete microbial genomes at NCBI currently contains only one fully annotated psychrotolerant archaeon (*Methanococcoides burtonii*). A proper explanation, therefore, awaits future investigation.

About aliphatic hydrophobic amino acids, we observe that Ala, Leu and Val are over-represented in thermophilic eubacteria, whereas in archaea only the beta branched amino acids, Ile, and to a lesser extent, Val, are over-represented. As already reported [12,18], thermo-labile Cys is under-represented in thermophiles of both archaeal and eubacterial origins. This suggests the possibility of a significant evolutionary pressure against Cys being conserved (assuming that thermophilic proteins evolved from mesophilic proteins) or being introduced (assuming that thermophilic proteins did not evolve from mesophilic proteins) *unless *it plays a structural (e.g., disulphide-bonded) or functional (e.g., metal-binding or catalytic) role. Trp is another potentially thermo-labile amino acid that is under-represented both in archaeal and eubacterial proteins.

### Environment specific amino acid composition

We inferred the secondary structure and the accessibility of the thermophilic proteins by aligning them to the mesophilic proteins of known structure. In Table 2 we report the amino acid compositions in the different environments considered. Strictly speaking, we show the amino acid composition of the regions of thermophilic proteins that were aligned against residues of the mesophilic homologues in alpha helix (HA, exposed or Ha, buried), in beta strand (EA, exposed or Ea), in coil (CA, exposed or Ca, buried) or with positive phi angles (PA, exposed or Pa, buried). The environments with the smallest differences between mesophilic and thermophilic proteins are PA and Pa, where the preference for Gly was very high and no large differences were observed for the other amino acids. For this reason the tables for residues with positive phi angles are not shown.

In general, the environments, in which we observed significant differences between thermophiles and mesophiles, are those exposed and in particular, exposed coils. In these environments, polar, non-charged amino acids are under-represented in thermophiles, whereas charged amino acids are over-represented.

Ion pairs stabilize proteins at high temperature more strongly than at low temperature [25-27] and desolvatation energy is lower for exposed charges than for buried ones [28]. We suggest that a large number of exposed charged amino acids can stabilise proteins at high temperatures, because they are able to form extended networks of ion pairs.

Below, we report several specific observations. In archaeal alpha helices, we observed a significant increase of Ile accompanied by a decrease of Ala on the exposed surface and a decrease of Leu on the buried surface.

On the surface of beta strands, we noticed that archaea prefer Ile and eubacteria prefer Val, both amino acids being beta branched. Ile is also over-represented on the buried side of beta strands.

There are contradictory reports concerning Pro. Some researchers observed that Pro has an increased occurrence in thermophilic proteins especially in loops [14,29,30]. Others [12,16] found that the frequency of Pro was unchanged. Our data show that the frequency of Pro does not change significantly in general, except for a minor, albeit significant increase in exposed loops of eubacteria.

### Environment specific substitution likelihoods

Amino acid composition can be a useful means to identify thermophilic organisms, but a more ambitious goal is to predict which substitutions are likely to change a mesophilic protein to a thermophilic one. The conservation of amino acid residues is strongly dependent on the environment in which they occur in the folded protein. Therefore, we calculated environment specific amino acid substitution likelihoods using a modified version of SUBST (K. Mizuguchi, unpublished). For each environment we calculated 20 × 20 substitution likelihoods. Each value represents the likelihood of occurrence and acceptance of a mutational event of a residue in the mesophilic sequence and in a particular structural environment, leading to any other residue in the thermophilic sequences. We compared these values with those representing the likelihood of occurrence and acceptance of a mutational event of a residue in the mesophilic sequence and in a particular structural environment, leading to any other residue in the mesophilic sequences. We show a list of statistically significant cases, in which the likelihood of a substitution leading from a mesophilic protein to a thermophilic archaeal protein or to a thermophilic eubacterial protein is different from the corresponding environment specific amino acid substitution in mesophilic proteins. For the sake of simplicity, in Table 3 (for archaea) and Table 4 (for eubacteria) we only show cases in which the difference is statistically significant (P < 0.01) and large (|Δ| > 2). All statistically significant cases are also provided in additional files 1 and 2.

As already observed in Table 2, major differences between thermophiles and mesophiles are observed in exposed environments. The substitutions that more frequently lead from mesophilic proteins to thermophilic proteins are those of polar, non-charged amino acids with Glu and Lys (in archaea) or with Arg (in eubacteria). In archaea, we also observe frequently the substitution of non-polar amino acids with Ile. The role of Ile is striking, since more than one third of the substitutions that lead from mesophilic to thermophilic archaeal proteins involve this amino acid. Substitutions of hydrophobic amino acids with Ile are highly frequent, in particular in the environment of exposed alpha helices. Ile is generally preferred to the gamma branched Leu, even in alpha helices and to the smaller beta branched Val. No hydrophobic amino acid has such prevalence in the case of eubacterial thermophilic proteins. Since the average nucleotidic composition does not differ significantly in the genomes of the archaea and eubacteria considered (Table 1), the abundance of Ile cannot be explained only by the fact that it is coded by triplets very rich in A/T (ATA, ATT and ATC).

## Conclusion

One reason to study naturally occurring thermostable proteins is to learn how mesophilic proteins of biotechnological interest can be stabilised. In this context, it is reassuring to observe that differences between thermophilic and mesophilic proteins occur primarily in solvent accessible surfaces. This suggests a possible strategy for enhancing the thermal stability of proteins: mutagenesis of exposed residues is in fact usually better tolerated by proteins, whereas mutagenesis of buried residues, even when rationally designed, can often lead to the misfolding of the protein of interest. By calculating the likelihood of substitutions that lead from mesophilic to thermophilic proteins, a simple and potentially useful trend for biotechnology was recognised in archaea, where polar, non-charged amino acids are preferentially substituted by Glu and Lys and non-polar amino acids by Ile.

Considering substitutions that lead from mesophilic to thermophilic proteins, we refer only to the fact that we aligned thermophilic proteins to their mesophilic homologues of known structure; by no means we want to imply that thermophilic proteins have evolutionarily derived from mesophilic proteins (or vice versa). Thermophiles are located at the deepest positions within the phylogenies of both prokaryotic domains. This observation led to the hypothesis of the hot origin of life but the matter is complex and still disputable [31]. Our data suggest that different strategies for thermal adaptation might have been exploited by archaea and eubacteria.

## Methods

Protein sequences for 10 thermophilic archaeal and seven thermophilic eubacterial genomes, as well as their GC content and optimal growth temperatures (OGTs), were obtained from the NCBI genome site [32]. These were the only thermophiles whose genomes had been completed and stored in this database at the time of investigation; we arbitrarily chose one species when two or more organisms belonging to the same genus were available.

The dataset of mesophilic structures was created from HOMSTRAD [19] available at HOMSTRAD site [33].

Each sequence in the dataset of mesophilic proteins was used as a query to search separately against the databases of thermophilic archaeal or eubacterial sequences. We performed gapped BLASTP searches in PSI-BLAST mode with the BLASTPGP [20] program using the following parameters: j, the maximum number of rounds was set to 2, h, the e-value threshold for including sequences in the score matrix model, was set to 0.000000001 and e, the final e-value was set to 0.000001. The same program produced the alignment of the query mesophilic sequence with its thermophilic homologues.

The first sequence of each alignment thus produced was a mesophilic protein of known 3D structure and its secondary structure/main chain conformational states and solvent accessibility were calculated by JOY [34]. Residues with side-chain relative accessibility higher than 7% were defined as accessible, otherwise inaccessible.

We modified (K. Mizuguchi, unpublished) the program SUBST available at SUBST site [35], which had been used to derive the environment specific substitution tables for the homology recognition software FUGUE [36]. The modified version of SUBST can now count amino acid substitutions between a protein of known structure and its homologous sequences. Observed amino acid replacements at aligned positions were counted in terms of the local environment of the first sequence (i.e., the mesophilic protein of known 3D structure). Let F^EN^_MT _be the number of times the amino acid M of the mesophilic protein in the environment EN was replaced in thermophilic proteins by the amino acid T. The raw substitution counts were converted into substitution frequencies ENMT as: ENMT= F^EN^_MT_/∑_t _F^EN^_Mt_. E generically refers to secondary structure/main chain conformation; specifically 'H' indicates alpha helices, 'E' beta strands, 'P' residues with a positive phi angle and 'C' coils. N generically refers to solvent accessibility; specifically 'A' indicates accessible side-chains and 'a' inaccessible side chains.

Two-tailed t-tests for independent samples were carried out to identify statistically significant (P < 0.01) differences between the values calculated for thermophilic proteins and the reference mesophilic proteins. The alignments built for archaeal proteins were randomly divided into four sets. For each set, environment specific amino acid compositions and substitutions were calculated. The means of these values were calculated to produce the final results. Similarly, the alignments built for eubacterial proteins and the control alignments of mesophilic proteins were each divided into four sets and the mean values of the amino acid compositions/substitutions were calculated. Differences between the means of two groups (e.g., thermophilic archaea and mesophiles) were then tested (with six degrees of freedom).

## Authors' contributions

MVC conceived the study. MVC and KM designed the experiments. KM provided coding and discussion on the methodology. MVC carried out the experiments. MVC and KM wrote the manuscript. MS helped in the elaboration of data.

## Supplementary Material

Additional file 1 Likelihoods of environment specific amino acid substitutions (in percent) that are significantly different between mesophiles-mesophiles and mesophiles-thermophilic archaeal homologues Click here for file

Additional file 2 Likelihoods of environment specific amino acid substitutions which are most biased in difference between mesophiles-mesophiles and mesophiles-thermophilic eubacterial homologues. Click here for file

## Figures and Tables

**Table 1:** G/C content and Optimal Growth Temperature of the organisms analysed in this paper.

**ARCHAEA**	**% G-C**	**OGT (°C)**
Aeropyrum pernix K1	56	90–95
Methanocaldococcus jannaschii DSM 2661	31	85
Methanothermobacter thermautotrophicus str. Delta_H	49	65–70
Archaeoglobus fulgidus DSM 4304	48	83
Thermoplasma acidophilum DSM 1728	45	59
Thermoplasma volcanium GSS1	39	60
Sulfolobus solfataricus P2	35	85
Pyrococcus furiosus DSM 3638	40	100
Methanopyrus kandleri AV19	61	98
Picrophilus torridus DSM 9790	35	60

**EUBACTERIA**	**% G-C**	**OGT (°C)**

Thermotoga maritima MSB8	46	80
Aquifex aeolicus VF5 96	43	96
Thermoanaerobacter tengcongensis MB4	37	75
Thermosynechococcus elongatus BP-1	53	55
Thermus thermophilus HB27	69	68
Geobacillus kaustophilus HTA426	52	55
Thermobifida fusca YX	67	50–55

Data were obtained from NCBI genome database [32] with the exception of G/C content and OGT of Geobacillus kaustophilus, which were taken from the DSMZ database of organisms (Braunschweg, Germany) [37].

**Table 2:** Environment specific amino acid compositions in percent.

	**Mes**	**t_arc**	**t_eu**		**mes**	**t_arc**	**t_bac**		**mes**	**t_arc**	**t_eu**
HAH	2.4	**1.7**	2.3	EAH	2.9	**1.8**	2.4	CAH	2.4	2.2	2.8
HAE	11.3	**14.5**	**14.2**	EAE	7.4	**9.8**	9.3	CAE	6.6	**8.9**	**8.6**
HAK	9.3	**10.6**	**8.1**	EAK	8.1	**10.3**	7.2	CAK	7.4	**8.5**	**6.5**
HAR	7.4	8.5	**10.1**	EAR	6.8	7.8	**9.2**	CAR	5.5	6.5	**7.1**
HAD	7.1	7.0	**5.9**	EAD	4.7	**5.8**	5.7	CAD	8.7	**9.6**	9.5
HAS	5.5	5.4	**3.9**	EAS	6.9	5.1	**4.1**	CAS	8.0	**7.2**	**6.0**
HAN	4.5	4.2	**2.9**	EAN	3.8	3.5	**2.5**	CAN	6.4	5.9	**4.5**
HAQ	5.8	**2.9**	**4.9**	EAQ	4.3	**2.0**	**3.1**	CAQ	3.9	**2.1**	**3.2**
HAC	0.7	**0.3**	**0.4**	EAC	1.4	0.4	0.5	CAC	1.2	**0.6**	**0.5**
HAT	4.4	**3.4**	**3.9**	EAT	9.4	**5.6**	7.0	CAT	7.1	**5.3**	**6.2**
HAP	3.2	3.0	3.6	EAP	3.0	3.3	3.9	CAP	8.2	7.6	**8.9**
HAA	9.9	**6.8**	10.6	EAA	4.6	4.0	5.4	CAA	6.6	**4.4**	6.5
HAG	3.5	3.6	3.7	EAG	3.4	3.6	3.9	CAG	6.4	6.4	6.8
HAI	3.7	**5.6**	3.8	EAI	5.6	**9.0**	6.2	CAI	3.1	**4.5**	3.3
HAV	4.4	4.7	4.8	EAV	8.9	10.0	**10.6**	CAV	4.5	4.7	4.7
HAL	7.5	7.5	8.3	EAL	6.1	5.7	7.7	CAL	5.3	5.6	**6.1**
HAM	1.9	**2.4**	1.8	EAM	1.6	1.8	1.4	CAM	1.5	**2.0**	1.7
HAF	2.8	2.7	2.7	EAF	4.1	3.7	3.7	CAF	2.9	3.0	2.8
HAY	3.3	**4.1**	3.1	EAY	5.5	5.8	5.0	CAY	3.3	**4.1**	3.2
HAW	1.3	1.0	1.1	EAW	1.7	1.0	1.2	CAW	1.1	0.9	0.9

	**mes**	**t_arc**	**t_eu**		**mes**	**t_arc**	**t_eu**		**mes**	**t_arc**	**t_eu**

HaH	1.8	**1.3**	1.5	EaH	1.6	1.2	1.2	CaH	2.8	2.3	2.7
HaE	2.1	**2.9**	2.7	EaE	1.6	1.7	1.6	CaE	2.5	3.0	2.8
HaK	1.5	2.1	1.7	EaK	1.1	**1.6**	1.3	CaK	1.6	**2.3**	1.9
HaR	2.2	2.3	2.5	EaR	1.7	1.6	1.7	CaR	2.2	2.5	2.6
HaD	2.0	2.2	1.9	EaD	2.0	2.1	2.1	CaD	4.3	3.8	4.1
HaS	3.9	4.6	3.3	EaS	3.8	3.5	**2.8**	CaS	6.5	6.4	**5.4**
HaN	1.8	1.8	1.6	EaN	1.8	1.7	1.6	CaN	3.7	3.4	2.8
HaQ	1.8	1.3	1.6	EaQ	1.5	**0.9**	**0.8**	CaQ	1.8	1.4	1.5
HaC	2.8	**1.0**	**1.3**	EaC	3.1	1.0	1.2	CaC	4.1	**1.7**	**1.6**
HaT	4.3	4.5	4.3	EaT	4.7	4.4	4.5	CaT	6.2	6.4	6.3
HaP	1.7	1.9	2.1	EaP	1.6	2.0	2.0	CaP	7.1	7.9	7.7
HaA	14.6	16.8	17.1	EaA	8.0	8.8	9.3	CaA	8.9	9.4	10.7
HaG	4.8	5.2	5.6	EaG	4.8	5.5	4.8	CaG	7.0	7.9	7.8
HaI	9.8	**12.2**	9.3	EaI	13.4	**18.5**	14.7	CaI	7.1	**9.2**	7.9
HaV	10.3	10.4	11.2	EaV	17.7	**20.8**	**22.9**	CaV	8.8	9.5	**10.0**
HaL	18.2	**15.8**	19.0	EaL	14.0	11.5	14.8	CaL	11.2	10.2	11.7
HaM	3.9	3.7	**3.3**	EaM	2.7	2.8	2.5	CaM	2.8	2.6	2.6
HaF	6.4	5.0	5.2	EaF	7.8	**5.5**	**5.4**	CaF	6.0	5.2	5.3
HaY	3.9	3.8	**3.2**	EaY	5.0	4.1	3.7	CaY	3.8	4.0	3.4
HaW	2.2	**1.0**	**1.4**	EaW	2.0	**0.8**	**1.1**	CaW	1.8	**1.0**	**1.2**

Mes stands for amino acid composition of mesophilic proteins, t_arc for amino acid composition of thermophilic archaeal proteins and t_eu for amino acid composition of thermophilic eubacterial proteins. HA stands for exposed alpha helices, Ha for non exposed alpha helices, EA for exposed beta strands, Ea for non exposed beta strands, CA for exposed coil and Ca for non exposed coil. The third letter is the standard code for amino acids. Values in bold significantly differ (P < 0.01) between thermophilic and mesophilic proteins.

**Table 3:** Likelihoods of environment specific amino acid substitutions (in percent) that are large and significantly different between mesophiles-mesophiles and mesophiles-thermophilic archaeal homologues.

**HA**			**Ha**			**EA**			**Ea**			**CA**			**Ca**		
**M→T**	**mes**	**t_arc**	**M→T**	**mes**	**t_arc**	**M→T**	**mes**	**t_arc**	**M→T**	**mes**	**t_arc**	**M→T**	**mes**	**t_arc**	**M→T**	**mes**	**t_arc**

*E→E*	*26,4*	*34,8*	*M→I*	*11,5*	*17,5*	*D→D*	*27,5*	*41,6*	*K→K*	*46,7*	*63,4*	*G→G*	*32,2*	*43,7*	*W→Y*	*5,7*	*11,2*
*I→I*	*16,5*	*23,6*	*F→I*	*6,4*	*11,5*	*I→I*	*18,3*	*30,5*	*I→I*	*30,5*	*38,2*	*E→E*	*19,4*	*28,9*	*L→I*	*10,6*	*14,8*
*D→E*	*15,8*	*22,0*	*L→I*	*12,3*	*16,6*	*N→N*	*13,8*	*22,9*	*F→I*	*9,2*	*16,3*	*D→D*	*29,7*	*38,7*	*C→V*	*1,5*	*5,4*
*Q→E*	*12,9*	*18,1*	*V→I*	*15,2*	*18,6*	*E→E*	*22,6*	*30,7*	*L→I*	*16,6*	*23,7*	*K→K*	*19,8*	*26,9*	*F→I*	*5,7*	*9,0*
*L→I*	*7,7*	*12,6*	*H→A*	*2,7*	*5,4*	*M→I*	*7,8*	*14,8*	*V→I*	*17,8*	*23,9*	*W→Y*	*7,7*	*14,0*			
*K→E*	*10,3*	*15,0*	*R→K*	*5,3*	*7,9*	*V→I*	*10,6*	*16,7*	*W→I*	*4,4*	*9,1*	*V→I*	*8,2*	*13,9*			
*V→I*	*9,2*	*13,5*	*W→I*	*3,6*	*6,0*	*Y→I*	*5,1*	*9,4*	*F→V*	*10,1*	*14,3*	*Q→E*	*8,9*	*13,7*			
*F→I*	*5,0*	*9,1*	*N→E*	*2,6*	*4,9*	*H→K*	*6,5*	*10,8*	*Y→I*	*6,0*	*10,0*	*L→I*	*6,9*	*11,5*			
*W→I*	*3,0*	*6,9*	**L→L**	**43,6**	**38,5**	*C→I*	*1,6*	*5,6*	*C→S*	*1,1*	*4,8*	*K→E*	*7,2*	*10,7*			
*N→E*	*9,4*	*12,8*				*F→I*	*7,6*	*11,4*	*T→V*	*11,9*	*15,3*	*D→E*	*8,1*	*11,4*			
*H→E*	*7,8*	*11,3*				*H→Y*	*5,9*	*9,6*	*H→Y*	*5,4*	*8,5*	*I→V*	*12,3*	*15,5*			
*M→I*	*7,9*	*11,1*				*M→Y*	*4,9*	*8,1*	**H→T**	**3,7**	**1,5**	*P→E*	*5,4*	*7,5*			
*A→E*	*10,3*	*13,2*				*T→K*	*7,7*	*9,8*	**Q→L**	**6,5**	**3,5**	**R→Q**	**4,4**	**2,4**			
*S→E*	*9,5*	*12,3*				**V→Q**	**3,0**	**1,0**	**I→L**	**18,6**	**13,6**	**V→T**	**7,4**	**5,4**			
*Q→K*	*10,3*	*12,9*				**P→L**	**4,8**	**2,7**				**T→Q**	**3,5**	**1,5**			
*E→K*	*8,6*	*11,3*				**N→L**	**4,1**	**1,9**				**N→T**	**6,5**	**4,5**			
*R→E*	*8,6*	*11,2*				**D→Q**	**3,4**	**1,1**				**R→T**	**5,3**	**3,3**			
*Y→I*	*4,0*	*6,3*				**A→Q**	**4,0**	**1,6**				**T→A**	**5,8**	**3,8**			
*N→R*	*6,3*	*8,3*				**T→Q**	**4,1**	**1,6**				**E→Q**	**5,0**	**2,9**			
**I→Q**	**3,2**	**1,2**				**D→S**	**7,2**	**4,6**				**P→T**	**4,9**	**2,8**			
**S→Q**	**4,9**	**2,8**				**N→Q**	**4,2**	**1,6**				**A→Q**	**4,0**	**1,9**			
**G→Q**	**4,3**	**2,1**				**H→Q**	**4,7**	**1,9**				**N→A**	**5,2**	**3,0**			
**L→A**	**7,8**	**5,7**				**R→Q**	**4,9**	**1,9**				**Q→A**	**6,3**	**4,1**			
**N→Q**	**5,5**	**2,9**				**K→Q**	**5,1**	**2,0**				**R→A**	**5,6**	**3,4**			
**V→A**	**11,5**	**8,9**				**I→T**	**7,0**	**3,8**				**E→T**	**5,6**	**3,3**			
**T→Q**	**5,2**	**2,5**				**A→T**	**8,6**	**5,3**				**H→A**	**5,2**	**2,8**			
**A→Q**	**5,2**	**2,3**				**D→T**	**7,1**	**3,4**				**K→T**	**5,9**	**3,4**			
**P→A**	**9,1**	**6,2**				**E→T**	**8,9**	**4,8**				**Q→T**	**6,3**	**3,8**			
**R→Q**	**5,8**	**2,9**				**N→T**	**9,8**	**5,3**				**K→A**	**5,9**	**3,4**			
**D→Q**	**5,7**	**2,8**										**D→A**	**4,8**	**2,2**			
**R→A**	**7,7**	**4,6**										**K→Q**	**4,8**	**2,1**			
**T→A**	**10,3**	**7,1**										**M→A**	**6,2**	**3,5**			
**K→Q**	**6,3**	**3,1**										**E→A**	**5,9**	**2,8**			
**E→Q**	**6,5**	**3,1**										**P→A**	**6,6**	**3,4**			
**N→A**	**8,6**	**4,9**															
**K→A**	**8,8**	**4,5**															
**Q→A**	**9,2**	**4,8**															
**D→A**	**8,1**	**3,6**															
**E→A**	**8,9**	**4,2**															
**C→C**	**74,4**	**36,9**															

Mes stands for mesophilic proteins, t_arc for thermophilic archaeal proteins, HA for exposed alpha helices, Ha for non exposed alpha helices, EA for exposed beta strands, Ea for non exposed beta strands, CA for exposed coil and Ca for non exposed coil. Data are shown only if P < 0.01 in the two-tailed t-test and if the difference between mes and t_arc are, in absolute value, larger than 2. Environment specific amino acid substitutions with higher likelihood values in mesophiles-thermophilic than in archaeal homologues are in italics, those with higher likelihood values in mesophiles-mesophiles homologues are in bold. Data are sorted by increasing differences between mes and t_arc.

**Table 4:** Likelihoods of environment-specific amino acid substitutions (in percent) that are large and significantly different between mesophiles-mesophiles and mesophiles-thermophilic eubacterial homologues.

**HA**			**Ha**			**EA**			**Ea**			**CA**			**Ca**		
**M→T**	**mes**	**t_eu**	**M→T**	**mes**	**t_eu**	**M→T**	**mes**	**t_eu**	**M→T**	**mes**	**t_eu**	**M→T**	**mes**	**t_eu**	**M→T**	**mes**	**t_eu**

*P→P*	*28,8*	*39,7*	*T→T*	*24,5*	*30,2*	*D→D*	*27,5*	*44,3*	*K→K*	*46,7*	*65,4*	*P→P*	*34,3*	*48,2*	*C→I*	*0,9*	*5,0*
*E→E*	*26,4*	*35,0*	*C→I*	*1,5*	*5,7*	*L→L*	*19,2*	*29,6*	*F→V*	*10,1*	*14,1*	*G→G*	*32,2*	*45,6*	*S→A*	*10,0*	*13,7*
*R→R*	*23,9*	*32,3*	*C→T*	*0,8*	*4,6*	*R→R*	*22,4*	*32,7*	**A→S**	**5,9**	**3,8**	*D→D*	*29,7*	*40,3*	*C→P*	*0,8*	*3,1*
*D→E*	*15,8*	*22,0*	*H→A*	*2,7*	*5,6*	*E→E*	*22,6*	*31,7*	**E→G**	**4,1**	**1,7**	*E→E*	*19,4*	*29,3*	**V→A**	**72,9**	**20,9**
*Q→E*	*12,9*	*17,9*				*V→V*	*22,9*	*31,1*	**C→C**	**67,7**	**25,3**	*R→R*	*21,8*	*31,0*			
*A→A*	*20,8*	*25,7*				*N→N*	*13,8*	*21,0*				*M→M*	*11,4*	*18,8*			
*K→R*	*10,7*	*15,4*				*K→R*	*10,2*	*16,1*				*C→A*	*1,9*	*8,9*			
*N→E*	*9,4*	*13,5*				*Q→E*	*9,6*	*15,1*				*V→V*	*17,6*	*23,8*			
*I→L*	*14,6*	*18,6*				*I→V*	*17,2*	*22,7*				*A→A*	*16,2*	*22,1*			
*V→V*	*15,8*	*19,5*				*C→L*	*2,1*	*6,0*				*Q→E*	*8,9*	*13,6*			
*N→R*	*6,3*	*9,9*				S→A	5,6	7,9				*K→R*	*8,4*	*13,0*			
*K→E*	*10,3*	*13,8*				**E→N**	**3,9**	**2,0**				*I→V*	*12,3*	*15,7*			
*F→L*	*12,0*	*15,4*				**A→T**	**8,6**	**5,9**				*V→I*	*8,2*	*11,1*			
*S→E*	*9,5*	*12,7*				**T→S**	**9,0**	**6,3**				*N→R*	*4,4*	*6,8*			
*Q→R*	*7,5*	*10,7*				**K→S**	**5,7**	**3,0**				*Q→R*	*6,1*	*8,5*			
*V→L*	*10,8*	*13,3*				**A→S**	**8,7**	**5,8**				*Y→L*	*6,0*	*8,1*			
*A→E*	*10,3*	*12,7*				**D→N**	**6,8**	**3,8**				*V→L*	*8,4*	*10,4*			
*T→R*	*5,7*	*7,9*				**H→S**	**6,5**	**3,1**				**I→N**	**3,4**	**1,5**			
*E→R*	*5,7*	*7,9*				**D→T**	**7,1**	**3,5**				**V→S**	**5,2**	**3,1**			
*S→R*	*5,6*	*7,7*				**D→S**	**7,2**	**3,6**				**E→S**	**6,9**	**4,8**			
*D→R*	*5,0*	*7,1*										**F→N**	**3,6**	**1,5**			
**Q→N**	**4,8**	**2,8**										**A→N**	**5,1**	**3,0**			
**D→N**	**5,2**	**3,2**										**Q→N**	**5,9**	**3,8**			
**T→S**	**7,8**	**5,8**										**R→S**	**6,1**	**3,9**			
**M→K**	**6,9**	**4,7**										**K→S**	**6,4**	**4,2**			
**G→S**	**7,6**	**5,5**										**M→N**	**4,3**	**2,1**			
**A→S**	**7,2**	**4,9**										**L→S**	**4,7**	**2,4**			
**G→D**	**6,7**	**4,4**										**E→N**	**5,4**	**3,1**			
**D→S**	**6,1**	**3,7**										**K→N**	**5,8**	**3,5**			
**P→S**	**6,3**	**3,2**										**Q→S**	**7,2**	**5,0**			
**C→C**	**74,4**	**26,4**										**T→N**	**6,2**	**3,9**			
												**I→K**	**5,1**	**2,8**			
												**D→S**	**7,4**	**5,0**			
												**P→S**	**6,3**	**3,9**			
												**M→S**	**5,3**	**2,8**			
												**D→N**	**8,6**	**6,0**			
												**H→N**	**7,7**	**5,0**			
												**G→N**	**5,8**	**2,9**			
												**C→C**	**71,4**	**22,8**			

Mes stands for mesophilic proteins, t_eu for thermophilic eubacterial proteins, HA for exposed alpha helices, Ha for non exposed alpha helices, EA for exposed beta strands, Ea for non exposed beta strands, CA for exposed coil and Ca for non exposed coil. Data are shown only if P < 0.01 in the two-tailed t-test and if the difference between mes and t_eu are, in absolute value, larger than 2. Environment specific amino acid substitutions with higher likelihood values in mesophiles-thermophilic than in eubacterial homologues are in italics, those with higher likelihood values in mesophiles-mesophiles homologues are in bold. Data are sorted by increasing differences between mes and t_eu.
