# Supplementary material for: Environment specific substitution tables for thermophilic proteins
Source: BMC Bioinformatics. 2007 Mar 8;8(Suppl 1):S15. doi: 10.1186/1471-2105-8-S1-S15 (PMC1885844; doi:10.1186/1471-2105-8-S1-S15)
Supplement: Additional file 1 — Likelihoods of environment specific amino acid substitutions (in percent) that are significantly different between mesophiles-mesophiles and mesophiles-thermophilic archaeal homologues [file 1471-2105-8-S1-S15-S1.doc]

ADDITIONAL FILE 1

Likelihoods of environment-specific amino acid substitutions (in percent) that are significantly different between mesophiles-mesophiles and mesophiles-thermophilic archaeal homologues.

|  | **mes** | **T_arc** |
| --- | --- | --- |
| EaKK | 46.7 | 63.4 |
| EADD | 27.5 | 41.6 |
| EAII | 18.3 | 30.5 |
| CAGG | 32.2 | 43.7 |
| CAEE | 19.4 | 28.9 |
| EANN | 13.8 | 22.9 |
| CADD | 29.7 | 38.7 |
| HAEE | 26.4 | 34.8 |
| EAEE | 22.6 | 30.7 |
| EaII | 30.5 | 38.2 |
| EaFI | 9.2 | 16.3 |
| CAKK | 19.8 | 26.9 |
| HAII | 16.5 | 23.6 |
| EAMI | 7.8 | 14.8 |
| EaLI | 16.6 | 23.7 |
| HADE | 15.8 | 22.0 |
| CAWY | 7.7 | 14.0 |
| EAVI | 10.6 | 16.7 |
| EaVI | 17.8 | 23.9 |
| HaMI | 11.5 | 17.5 |
| CAVI | 8.2 | 13.9 |
| CaWY | 5.7 | 11.2 |
| HAQE | 12.9 | 18.1 |
| HaFI | 6.4 | 11.5 |
| HALI | 7.7 | 12.6 |
| CAQE | 8.9 | 13.7 |
| HAKE | 10.3 | 15.0 |
| EaWI | 4.4 | 9.1 |
| CALI | 6.9 | 11.5 |
| HAVI | 9.2 | 13.5 |
| HaLI | 12.3 | 16.6 |
| EAYI | 5.1 | 9.4 |
| EAHK | 6.5 | 10.8 |
| CaLI | 10.6 | 14.8 |
| EaFV | 10.1 | 14.3 |
| HAFI | 5.0 | 9.1 |
| EACI | 1.6 | 5.6 |
| EaYI | 6.0 | 10.0 |
| HAWI | 3.0 | 6.9 |
| CaCV | 1.5 | 5.4 |
| EAFI | 7.6 | 11.4 |
| EaCS | 1.1 | 4.8 |
| EAHY | 5.9 | 9.6 |
| CAKE | 7.2 | 10.7 |
| HANE | 9.4 | 12.8 |
| HAHE | 7.8 | 11.3 |
| EaTV | 11.9 | 15.3 |
| HaVI | 15.2 | 18.6 |
| CADE | 8.1 | 11.4 |
| HAMI | 7.9 | 11.1 |
| CaFI | 5.7 | 9.0 |
| EAMY | 4.9 | 8.1 |
| CAIV | 12.3 | 15.5 |
| EaHY | 5.4 | 8.5 |
| HAAE | 10.3 | 13.2 |
| HASE | 9.5 | 12.3 |
| HaHA | 2.7 | 5.4 |
| HAQK | 10.3 | 12.9 |
| HARE | 8.6 | 11.2 |
| HAEK | 8.6 | 11.3 |
| HaRK | 5.3 | 7.9 |
| HaWI | 3.6 | 6.0 |
| HAYI | 4.0 | 6.3 |
| HaNE | 2.6 | 4.9 |
| CAPE | 5.4 | 7.5 |
| EATK | 7.7 | 9.8 |
| HANR | 6.3 | 8.3 |
| HaFV | 6.0 | 7.9 |
| CAHY | 4.6 | 6.5 |
| HAQR | 7.5 | 9.3 |
| CAYI | 2.9 | 4.7 |
| HAAI | 2.6 | 4.4 |
| HAKR | 10.7 | 12.4 |
| CaFV | 5.2 | 7.0 |
| CATI | 2.4 | 4.2 |
| CANR | 4.4 | 5.9 |
| HaAS | 5.7 | 7.2 |
| HADK | 7.6 | 9.1 |
| HaWM | 2.2 | 3.7 |
| CALM | 3.3 | 4.7 |
| CAED | 10.5 | 11.8 |
| CARE | 5.7 | 7.0 |
| HAAY | 1.9 | 3.1 |
| HAER | 5.7 | 6.9 |
| EAQI | 3.6 | 4.8 |
| HaIP | 0.9 | 0.9 |
| CaSK | 2.0 | 3.1 |
| CAHD | 5.8 | 6.9 |
| CAAI | 2.7 | 3.8 |
| HAQI | 2.3 | 3.3 |
| CAYL | 6.0 | 6.8 |
| HAQY | 2.1 | 2.9 |
| HAKI | 2.4 | 3.2 |
| CATY | 1.9 | 2.7 |
| HaAE | 1.3 | 2.1 |
| HAYM | 1.9 | 2.6 |
| HAEC | 0.3 | 0.1 |
| HAWC | 0.4 | 0.2 |
| HaLH | 0.7 | 0.4 |
| HADC | 0.3 | 0.1 |
| HAQC | 0.4 | 0.1 |
| CANC | 0.6 | 0.3 |
| CAKF | 1.6 | 1.2 |
| CARC | 0.6 | 0.3 |
| CAEC | 0.5 | 0.2 |
| HALC | 0.7 | 0.3 |
| HAVC | 0.8 | 0.4 |
| CAGC | 0.8 | 0.3 |
| HaAW | 0.8 | 0.3 |
| HADL | 3.2 | 2.7 |
| HaIW | 1.0 | 0.5 |
| HAIP | 2.2 | 1.7 |
| CAIH | 1.5 | 0.9 |
| HAMC | 0.8 | 0.3 |
| EAKW | 0.9 | 0.3 |
| EADW | 0.7 | 0.2 |
| CARH | 2.5 | 2.0 |
| CADH | 1.8 | 1.2 |
| HALH | 1.6 | 1.0 |
| HAAH | 1.7 | 1.1 |
| EAVW | 1.0 | 0.4 |
| EAKC | 0.8 | 0.2 |
| CAEH | 2.0 | 1.4 |
| EADC | 0.7 | 0.1 |
| CaFQ | 1.2 | 0.6 |
| HAMH | 1.5 | 0.9 |
| HaLC | 1.2 | 0.6 |
| HAAP | 3.1 | 2.5 |
| HARP | 1.9 | 1.2 |
| EaAQ | 1.2 | 0.5 |
| EANM | 1.3 | 0.6 |
| EALC | 1.1 | 0.4 |
| EAEC | 0.8 | 0.2 |
| HAEH | 1.9 | 1.2 |
| CAKH | 2.2 | 1.5 |
| EaPC | 1.1 | 0.4 |
| EAKN | 4.0 | 3.2 |
| HADH | 2.0 | 1.2 |
| HAKH | 2.2 | 1.5 |
| CADL | 2.6 | 1.8 |
| HALT | 3.6 | 2.8 |
| EATH | 2.2 | 1.3 |
| CAVH | 1.7 | 0.9 |
| HATH | 2.1 | 1.2 |
| CAFE | 3.7 | 2.8 |
| EaIC | 1.4 | 0.5 |
| HaMC | 1.4 | 0.5 |
| HAID | 2.9 | 2.0 |
| CAEV | 3.4 | 2.5 |
| EAIN | 2.1 | 1.1 |
| EAPQ | 2.8 | 1.9 |
| HAQH | 2.6 | 1.5 |
| EAKH | 2.6 | 1.6 |
| EAVC | 1.4 | 0.3 |
| HaGQ | 1.6 | 0.5 |
| HaDF | 1.8 | 0.7 |
| HASH | 2.2 | 1.2 |
| CAYS | 4.6 | 3.5 |
| EAQC | 1.3 | 0.2 |
| HaTC | 2.0 | 1.0 |
| CAID | 3.3 | 2.2 |
| EAVH | 1.8 | 0.7 |
| EaTQ | 1.9 | 0.8 |
| HADP | 3.3 | 2.1 |
| EAIG | 1.9 | 0.8 |
| HaAC | 2.3 | 1.1 |
| EARH | 2.8 | 1.6 |
| HAAT | 4.6 | 3.4 |
| HADT | 3.5 | 2.3 |
| EaTC | 2.5 | 1.3 |
| CaFW | 3.6 | 2.4 |
| CATP | 5.4 | 4.2 |
| HART | 3.5 | 2.2 |
| CAYQ | 2.6 | 1.3 |
| HANT | 4.5 | 3.2 |
| EAYH | 2.8 | 1.5 |
| CALN | 3.4 | 2.1 |
| HAKT | 3.7 | 2.3 |
| CAAT | 6.1 | 4.7 |
| CAIN | 3.4 | 2.0 |
| HAFA | 6.4 | 4.9 |
| EAEL | 3.9 | 2.4 |
| CAPS | 6.3 | 4.8 |
| CALD | 3.8 | 2.3 |
| CAVN | 4.0 | 2.4 |
| CANQ | 3.5 | 1.9 |
| CaPA | 6.0 | 4.4 |
| CAHQ | 3.9 | 2.3 |
| CAST | 9.5 | 7.9 |
| CAFQ | 2.6 | 1.0 |
| EAYQ | 2.6 | 1.0 |
| CAPQ | 2.8 | 1.2 |
| CADS | 7.4 | 5.8 |
| CALQ | 3.1 | 1.5 |
| CAEP | 6.1 | 4.4 |
| CARP | 5.3 | 3.7 |
| CAIQ | 2.7 | 1.0 |
| HAET | 3.8 | 2.1 |
| EADL | 3.0 | 1.3 |
| CADQ | 3.2 | 1.5 |
| EALQ | 2.9 | 1.2 |
| CADT | 5.2 | 3.5 |
| HAYQ | 3.5 | 1.8 |
| CAVG | 4.4 | 2.7 |
| CASA | 7.0 | 5.2 |
| HAVQ | 3.4 | 1.6 |
| EaQT | 4.6 | 2.8 |
| EAAS | 8.7 | 6.8 |
| CAGT | 5.0 | 3.2 |
| HAFQ | 2.8 | 1.0 |
| CAIP | 5.3 | 3.5 |
| CAMQ | 3.7 | 1.9 |
| CASQ | 3.6 | 1.7 |
| HALQ | 3.3 | 1.4 |
| HAPQ | 3.6 | 1.7 |
| EAIF | 5.7 | 3.8 |
| CAEG | 6.4 | 4.5 |
| CAFA | 4.8 | 2.8 |
| CAVQ | 3.2 | 1.3 |
| CAGQ | 3.2 | 1.3 |
| CARQ | 4.4 | 2.4 |
| HAIQ | 3.2 | 1.2 |
| CAVT | 7.4 | 5.4 |
| EAVQ | 3.0 | 1.0 |
| CATQ | 3.5 | 1.5 |
| CANT | 6.5 | 4.5 |
| CART | 5.3 | 3.3 |
| CATA | 5.8 | 3.8 |
| EAPL | 4.8 | 2.7 |
| CAEQ | 5.0 | 2.9 |
| HASQ | 4.9 | 2.8 |
| CAPT | 4.9 | 2.8 |
| CAAQ | 4.0 | 1.9 |
| EANL | 4.1 | 1.9 |
| EaHT | 3.7 | 1.5 |
| HAGQ | 4.3 | 2.1 |
| HALA | 7.8 | 5.7 |
| CANA | 5.2 | 3.0 |
| CAQA | 6.3 | 4.1 |
| CARA | 5.6 | 3.4 |
| EADQ | 3.4 | 1.1 |
| CAET | 5.6 | 3.3 |
| CAHA | 5.2 | 2.8 |
| EAAQ | 4.0 | 1.6 |
| CAKT | 5.9 | 3.4 |
| EATQ | 4.1 | 1.6 |
| CAQT | 6.3 | 3.8 |
| CAKA | 5.9 | 3.4 |
| CADA | 4.8 | 2.2 |
| HANQ | 5.5 | 2.9 |
| HAVA | 11.5 | 8.9 |
| EADS | 7.2 | 4.6 |
| EANQ | 4.2 | 1.6 |
| CAKQ | 4.8 | 2.1 |
| CAMA | 6.2 | 3.5 |
| EAHQ | 4.7 | 1.9 |
| HATQ | 5.2 | 2.5 |
| HAAQ | 5.2 | 2.3 |
| HAPA | 9.1 | 6.2 |
| HARQ | 5.8 | 2.9 |
| EARQ | 4.9 | 1.9 |
| HADQ | 5.7 | 2.8 |
| EaQL | 6.5 | 3.5 |
| HARA | 7.7 | 4.6 |
| EAKQ | 5.1 | 2.0 |
| EAIT | 7.0 | 3.8 |
| CAEA | 5.9 | 2.8 |
| HATA | 10.3 | 7.1 |
| CAPA | 6.6 | 3.4 |
| HAKQ | 6.3 | 3.1 |
| EAAT | 8.6 | 5.3 |
| HAEQ | 6.5 | 3.1 |
| EADT | 7.1 | 3.4 |
| HANA | 8.6 | 4.9 |
| EAET | 8.9 | 4.8 |
| HAKA | 8.8 | 4.5 |
| HAQA | 9.2 | 4.8 |
| HADA | 8.1 | 3.6 |
| EANT | 9.8 | 5.3 |
| HAEA | 8.9 | 4.2 |
| EaIL | 18.6 | 13.6 |
| HaLL | 43.6 | 38.5 |
| HACC | 74.4 | 36.9 |

In the first column, the first character is ‘H’ for alpha helices, ‘E’ for beta strands, ‘C’ for coils; the second character is ‘A’ for accessible, ‘a’ for inaccessible; the third character is the amino acid from which we observe the substitution; the fourth character is the amino acid with which we observe the substitution in mesophilic proteins (mes) or thermophilic archaeal proteins (t_arc). Data are shown only if P<0.01 in the two-tailed t-test. Environment-specific amino acid substitutions with higher likelihood values in mesophiles-thermophilic archaeal homologues are in red, those with higher likelihood values in mesophiles- mesophiles homologues are in blue. Data are sorted by increasing differences between mes and t_arc.
