# Supplementary material for: Environment specific substitution tables for thermophilic proteins
Source: BMC Bioinformatics. 2007 Mar 8;8(Suppl 1):S15. doi: 10.1186/1471-2105-8-S1-S15 (PMC1885844; doi:10.1186/1471-2105-8-S1-S15)
Supplement: Additional file 2 — Likelihoods of environment specific amino acid substitutions which are most biased in difference between mesophiles-mesophiles and mesophiles-thermophilic eubacterial homologues. [file 1471-2105-8-S1-S15-S2.doc]

ADDITIONAL FILE 2

Probabilities of environment specific amino acid substitutions which are most biased in difference between mesophiles-mesophiles and mesophiles- thermophilic eubacterial homologues.

|  | **mes** | **T_eu** |
| --- | --- | --- |
| EaKK | 46.7 | 65.4 |
| EADD | 27.5 | 44.3 |
| CAPP | 34.3 | 48.2 |
| CAGG | 32.2 | 45.6 |
| HAPP | 28.8 | 39.7 |
| CADD | 29.7 | 40.3 |
| EALL | 19.2 | 29.6 |
| EARR | 22.4 | 32.7 |
| CAEE | 19.4 | 29.3 |
| CARR | 21.8 | 31.0 |
| EAEE | 22.6 | 31.7 |
| HAEE | 26.4 | 35.0 |
| HARR | 23.9 | 32.3 |
| EAVV | 22.9 | 31.1 |
| CAMM | 11.4 | 18.8 |
| EANN | 13.8 | 21.0 |
| CACA | 1.9 | 8.9 |
| HADE | 15.8 | 22.0 |
| CAVV | 17.6 | 23.8 |
| CAAA | 16.2 | 22.1 |
| EAKR | 10.2 | 16.1 |
| HaTT | 24.5 | 30.2 |
| EAQE | 9.6 | 15.1 |
| EAIV | 17.2 | 22.7 |
| HAQE | 12.9 | 17.9 |
| HAAA | 20.8 | 25.7 |
| HAKR | 10.7 | 15.4 |
| CAQE | 8.9 | 13.6 |
| CAKR | 8.4 | 13.0 |
| HANE | 9.4 | 13.5 |
| HaCI | 1.5 | 5.7 |
| CaCI | 0.9 | 5.0 |
| HAIL | 14.6 | 18.6 |
| EaFV | 10.1 | 14.1 |
| EACL | 2.1 | 6.0 |
| HaCT | 0.8 | 4.6 |
| CaSA | 10.0 | 13.7 |
| HAVV | 15.8 | 19.5 |
| HANR | 6.3 | 9.9 |
| HAKE | 10.3 | 13.8 |
| CAIV | 12.3 | 15.7 |
| HAFL | 12.0 | 15.4 |
| HASE | 9.5 | 12.7 |
| HAQR | 7.5 | 10.7 |
| HaHA | 2.7 | 5.6 |
| CAVI | 8.2 | 11.1 |
| CANR | 4.4 | 6.8 |
| HAVL | 10.8 | 13.3 |
| CAQR | 6.1 | 8.5 |
| HAAE | 10.3 | 12.7 |
| HATR | 5.7 | 7.9 |
| CaCP | 0.8 | 3.1 |
| HAER | 5.7 | 7.9 |
| EASA | 5.6 | 7.9 |
| HASR | 5.6 | 7.7 |
| HADR | 5.0 | 7.1 |
| CAYL | 6.0 | 8.1 |
| CAVL | 8.4 | 10.4 |
| HAAR | 5.4 | 7.3 |
| HaVA | 11.6 | 13.4 |
| HAWF | 5.3 | 7.1 |
| EAGA | 7.1 | 8.8 |
| HARE | 8.6 | 10.2 |
| CALI | 6.9 | 8.4 |
| CaVA | 7.8 | 9.3 |
| CASE | 6.0 | 7.2 |
| CAYR | 3.6 | 4.8 |
| CaCH | 0.3 | 1.3 |
| CARE | 5.7 | 6.7 |
| HaAE | 1.3 | 1.8 |
| HAEC | 0.3 | 0.1 |
| HADC | 0.3 | 0.1 |
| HAQC | 0.4 | 0.2 |
| HAAH | 1.7 | 1.5 |
| HaVK | 1.0 | 0.8 |
| EaIN | 0.5 | 0.3 |
| CARC | 0.6 | 0.3 |
| CADF | 1.2 | 0.9 |
| HALC | 0.7 | 0.3 |
| CAEC | 0.5 | 0.2 |
| CANI | 1.6 | 1.3 |
| CAGC | 0.8 | 0.4 |
| HAQI | 2.3 | 1.9 |
| HAVC | 0.8 | 0.4 |
| CAKF | 1.6 | 1.2 |
| CaVK | 1.3 | 0.9 |
| CAEW | 0.8 | 0.4 |
| EAKC | 0.8 | 0.4 |
| CAMC | 0.9 | 0.5 |
| CaSW | 0.9 | 0.4 |
| CAGW | 0.8 | 0.4 |
| CADI | 1.4 | 0.9 |
| EADW | 0.7 | 0.3 |
| CAEF | 1.6 | 1.1 |
| HaGN | 1.7 | 1.1 |
| EADC | 0.7 | 0.2 |
| EAEC | 0.8 | 0.3 |
| EAEF | 1.8 | 1.2 |
| HALT | 3.6 | 3.0 |
| EaAQ | 1.2 | 0.5 |
| EAVH | 1.8 | 1.1 |
| CAPI | 2.3 | 1.6 |
| EANM | 1.3 | 0.5 |
| EAAD | 3.3 | 2.6 |
| CADL | 2.6 | 1.9 |
| HaGQ | 1.6 | 0.8 |
| EaIC | 1.4 | 0.6 |
| HALQ | 3.3 | 2.5 |
| HAKT | 3.7 | 2.9 |
| EaIT | 2.4 | 1.6 |
| EaKD | 1.1 | 0.3 |
| HaAC | 2.3 | 1.4 |
| CADQ | 3.2 | 2.4 |
| EAQC | 1.3 | 0.4 |
| HaYS | 2.2 | 1.4 |
| CaVN | 1.7 | 0.8 |
| EAYC | 1.4 | 0.6 |
| HAAT | 4.6 | 3.7 |
| EaTC | 2.5 | 1.6 |
| CAEV | 3.4 | 2.5 |
| HaTM | 3.2 | 2.3 |
| EaKN | 1.4 | 0.5 |
| CAVQ | 3.2 | 2.3 |
| CaPN | 2.0 | 1.1 |
| HAGQ | 4.3 | 3.3 |
| HADQ | 5.7 | 4.8 |
| HAFS | 3.0 | 2.0 |
| CAAT | 6.1 | 5.0 |
| CaFW | 3.6 | 2.6 |
| CAPQ | 2.8 | 1.8 |
| HALN | 2.4 | 1.4 |
| EAYN | 2.4 | 1.3 |
| EADQ | 3.4 | 2.3 |
| HAFN | 2.3 | 1.3 |
| HAIN | 2.4 | 1.4 |
| HAAQ | 5.2 | 4.2 |
| CATQ | 3.5 | 2.5 |
| CAIQ | 2.7 | 1.6 |
| CALQ | 3.1 | 2.0 |
| HaDN | 4.1 | 3.0 |
| CaVS | 2.8 | 1.7 |
| HaTC | 2.0 | 0.9 |
| HAEK | 8.6 | 7.5 |
| HAVD | 3.3 | 2.2 |
| CAGT | 5.0 | 3.8 |
| HAYS | 3.1 | 2.0 |
| HAMN | 2.8 | 1.7 |
| HaDF | 1.8 | 0.7 |
| CART | 5.3 | 4.2 |
| EAQN | 3.7 | 2.5 |
| HALD | 3.0 | 1.9 |
| EaTQ | 1.9 | 0.8 |
| CAPK | 5.7 | 4.5 |
| HAHS | 4.7 | 3.5 |
| EAIN | 2.1 | 0.9 |
| HAID | 2.9 | 1.6 |
| EALQ | 2.9 | 1.7 |
| HALS | 3.1 | 1.9 |
| EADL | 3.0 | 1.7 |
| EARN | 3.1 | 1.9 |
| EAYQ | 2.6 | 1.3 |
| EAIY | 4.4 | 3.1 |
| EAFN | 2.1 | 0.8 |
| HAIS | 3.4 | 2.1 |
| EANI | 3.2 | 2.0 |
| EAVN | 2.3 | 1.0 |
| HaAS | 5.7 | 4.4 |
| EaDV | 2.4 | 1.0 |
| CALK | 4.8 | 3.5 |
| HAVK | 5.8 | 4.5 |
| CaAC | 3.1 | 1.7 |
| HAYK | 5.0 | 3.5 |
| HARN | 3.9 | 2.5 |
| CAVT | 7.4 | 5.9 |
| HASN | 5.2 | 3.7 |
| HALK | 5.6 | 4.2 |
| CAFK | 3.5 | 2.1 |
| CAKT | 5.9 | 4.4 |
| EATK | 7.7 | 6.2 |
| CALD | 3.8 | 2.3 |
| HAKD | 6.0 | 4.5 |
| EAPQ | 2.8 | 1.3 |
| CAET | 5.6 | 4.1 |
| CAVD | 4.0 | 2.5 |
| HAVN | 3.0 | 1.5 |
| HAES | 5.0 | 3.4 |
| CADK | 5.6 | 4.0 |
| CAFS | 4.3 | 2.8 |
| EAKN | 4.0 | 2.4 |
| HAVS | 4.2 | 2.6 |
| HATN | 4.7 | 3.1 |
| HARD | 5.1 | 3.5 |
| CAPA | 6.6 | 5.0 |
| HAQD | 7.5 | 5.9 |
| EATN | 3.8 | 2.2 |
| HANS | 6.2 | 4.5 |
| HAIK | 5.5 | 3.9 |
| CAPT | 4.9 | 3.2 |
| CAIS | 4.3 | 2.6 |
| HaGS | 6.0 | 4.3 |
| CAQT | 6.3 | 4.5 |
| HAPN | 3.2 | 1.5 |
| HAKS | 5.1 | 3.4 |
| CAVK | 5.4 | 3.7 |
| CALN | 3.4 | 1.7 |
| EAHQ | 4.7 | 2.9 |
| HARS | 4.5 | 2.8 |
| CAYN | 4.0 | 2.2 |
| HAEN | 4.0 | 2.3 |
| HAAD | 6.0 | 4.2 |
| CAPN | 3.6 | 1.7 |
| CAHS | 6.0 | 4.1 |
| HAKN | 4.4 | 2.6 |
| HAGN | 4.6 | 2.7 |
| EAVS | 4.3 | 2.4 |
| HAAN | 4.1 | 2.2 |
| HAED | 10.3 | 8.4 |
| HASK | 8.2 | 6.3 |
| CAVN | 4.0 | 2.0 |
| EAEN | 3.9 | 2.0 |
| CAIN | 3.4 | 1.5 |
| HAQN | 4.8 | 2.8 |
| HADN | 5.2 | 3.2 |
| HATS | 7.8 | 5.8 |
| CAVS | 5.2 | 3.1 |
| CAES | 6.9 | 4.8 |
| CAFN | 3.6 | 1.5 |
| EaAS | 5.9 | 3.8 |
| CAAN | 5.1 | 3.0 |
| CAQN | 5.9 | 3.8 |
| HAMK | 6.9 | 4.7 |
| HAGS | 7.6 | 5.5 |
| CARS | 6.1 | 3.9 |
| CAKS | 6.4 | 4.2 |
| CAMN | 4.3 | 2.1 |
| HAAS | 7.2 | 4.9 |
| CALS | 4.7 | 2.4 |
| CAEN | 5.4 | 3.1 |
| CAQS | 7.2 | 5.0 |
| CAKN | 5.8 | 3.5 |
| CATN | 6.2 | 3.9 |
| CAIK | 5.1 | 2.8 |
| HAGD | 6.7 | 4.4 |
| CADS | 7.4 | 5.0 |
| HADS | 6.1 | 3.7 |
| EaEG | 4.1 | 1.7 |
| CAPS | 6.3 | 3.9 |
| CAMS | 5.3 | 2.8 |
| CADN | 8.6 | 6.0 |
| EAAT | 8.6 | 5.9 |
| EATS | 9.0 | 6.3 |
| EAKS | 5.7 | 3.0 |
| CAHN | 7.7 | 5.0 |
| CAGN | 5.8 | 2.9 |
| EAAS | 8.7 | 5.8 |
| EADN | 6.8 | 3.8 |
| HAPS | 6.3 | 3.2 |
| EAHS | 6.5 | 3.1 |
| EADT | 7.1 | 3.5 |
| EADS | 7.2 | 3.6 |
| EaCC | 67.7 | 25.3 |
| HACC | 74.4 | 26.4 |
| CACC | 71.4 | 22.8 |
| CaCC | 72.9 | 20.9 |

.

In the first column, the first character is ‘H’ for alpha helices, ‘E’ for beta strands, ‘C’ for coils; the second character is ‘A’ for accessible, ‘a’ for inaccessible; the third character is the amino acid from which we observe the substitution; the fourth character is the amino acid with which we observe the substitution in mesophilic proteins (mes) or thermophilic

old: archaeal

new: eubacterial

proteins (t_eu). Data are shown only if P<0.01 in the two-tailed t-test. Environment-specific amino acid substitutions with higher likelihood values in mesophiles-thermophilic eubacterial homologues are in red, those with higher likelihood values in mesophiles- mesophiles homologues are in blue. Data are sorted by increasing differences between mes and t_eu.
